# Supplementary material for: Human respiratory organoids sustained reproducible propagation of human rhinovirus C and elucidation of virus-host interaction
Source: Nat Commun. 2024 Dec 30;15:10772. doi: 10.1038/s41467-024-55076-2 (PMC11686133; doi:10.1038/s41467-024-55076-2)
Supplement: Supplementary file 1 — Supplementary Information file [file 41467_2024_55076_MOESM1_ESM.pdf]

Supplementary Figure 1. Phylogenetic analysis of 8 isolated HRV-C and 43 HRV-C reference subtypes based on the 5′ UTR (372bp, position nt 179-551).

5‘ UTR

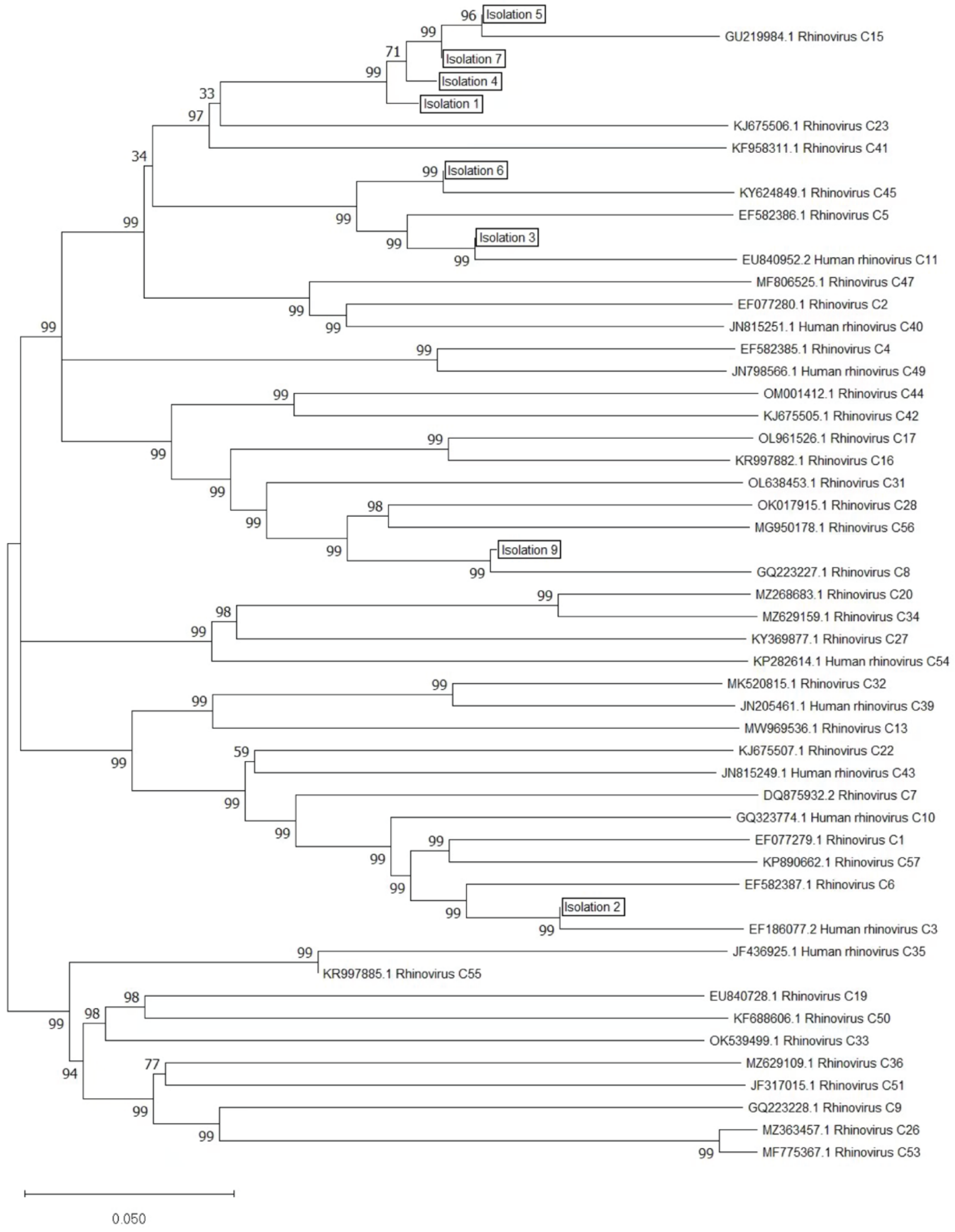

The phylogenetic tree was constructed using the neighbor-joining method and p-distance model with bootstrap replicated from 1,000 trees using MEGA 10.2.3. Genetic distance is represented by the scale bar.

Supplementary Figure 2. Phylogenetic analysis of 8 isolated HRV-C and 43 HRV-C reference subtypes based on the VP4/VP2 gene (540bp, position nt 547-1087).

VP4/VP2

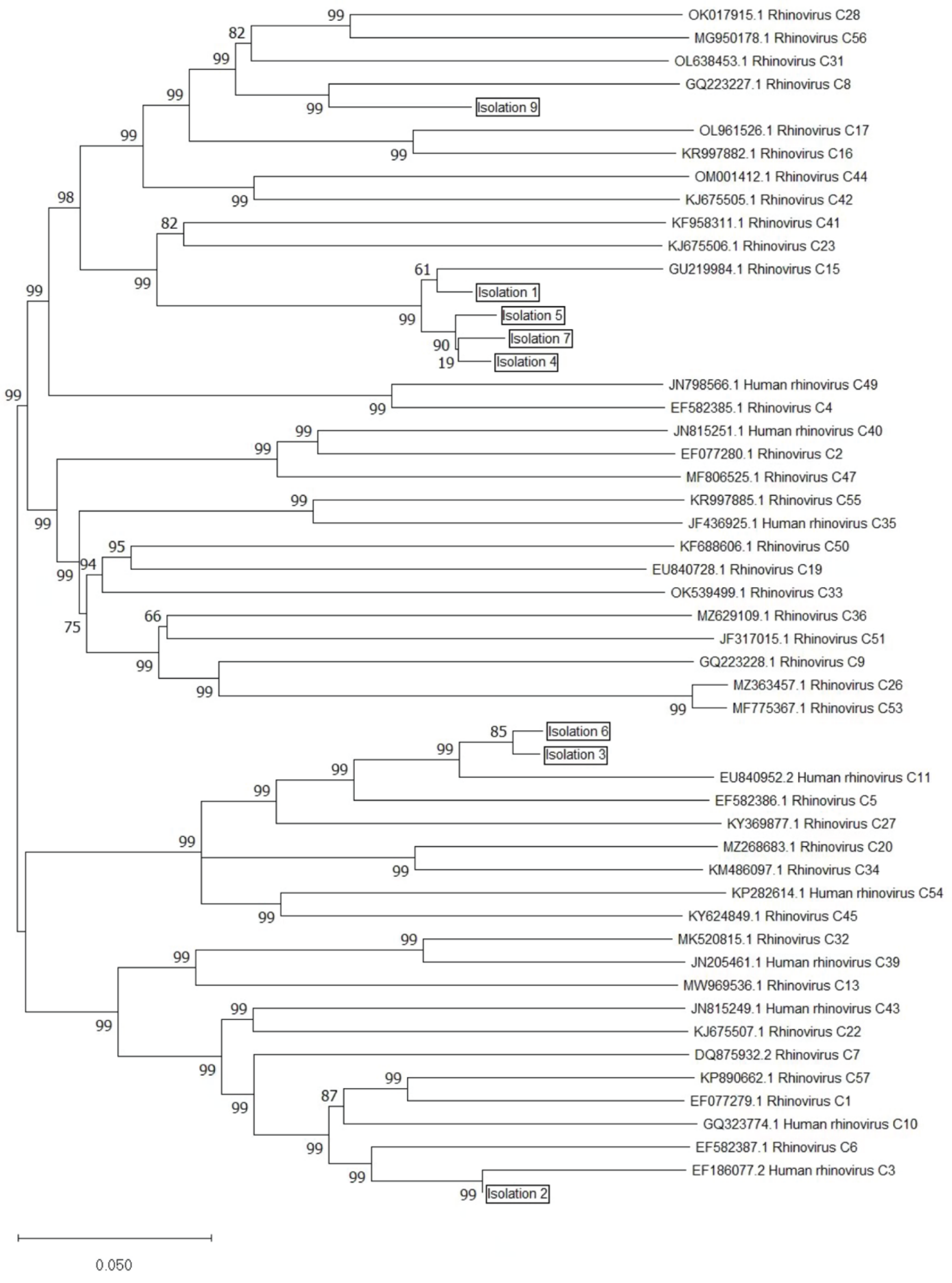

The phylogenetic tree was constructed using the neighbor-joining method and p-distance model with bootstrap replicated from 1,000 trees using MEGA 10.2.3. Genetic distance is represented by the scale bar.

Supplementary Figure 3. Orthogonal views of human airway organoid with HRV-C infection.

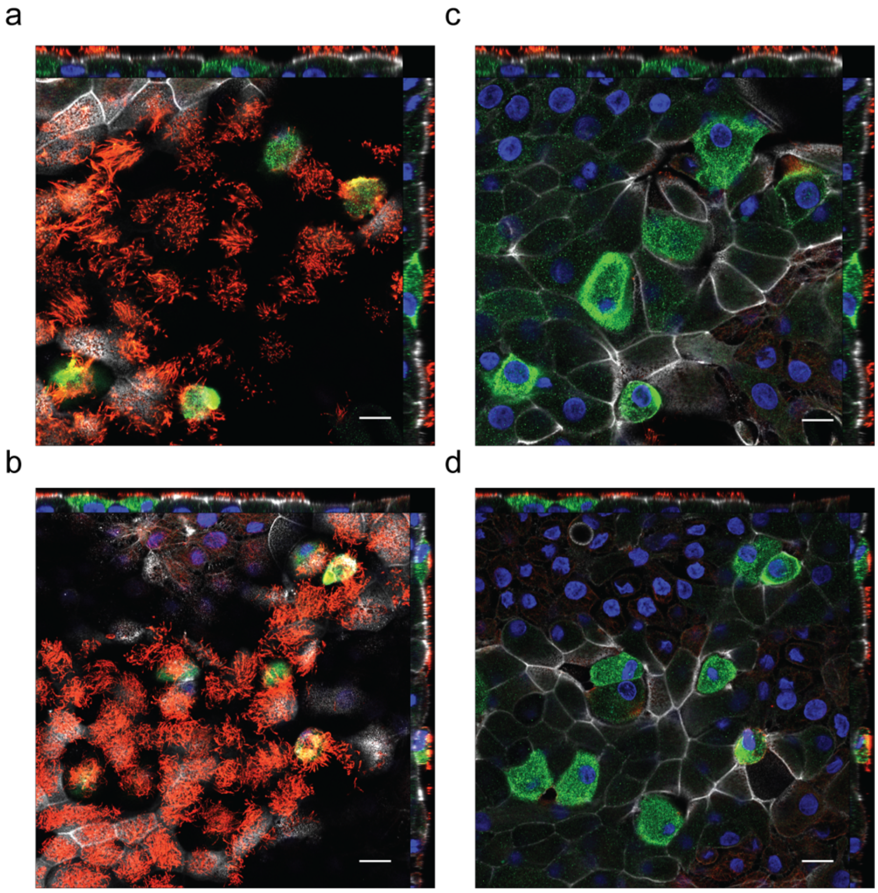

(a-d) Orthogonal views of human airway organoid with HRV-C infection. Airway organoids were fixed and double-stained with an α-VP3 (green) and α-ACCTUB (red). Upper layers (a, b) and lower layers (c, d) of orthogonal views of confocal images with en face and cross-section are shown. Nuclei and actin filaments were counterstained with DAPI (blue) and Phalloidin-647 (white), respectively. The experiment was performed three times independently with similar results. Scale bar, 10 µm.

Supplementary Figure 4. Human nasal organoid sustained serial HRV-C propagation.

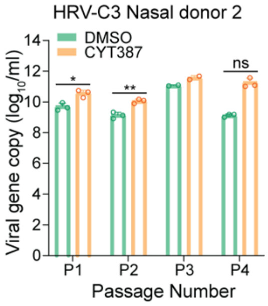

Nasal organoids pre-treated with CYT387 or DMSO were inoculated with HRV-C3 at 100 viral gene copy/cell and incubated in the presence of CYT387 or DMSO respectively. CYT387- and DMSO-treated media were brought forward as inoculum to the next round of infection, during which CYT387 and DMSO treatment continued. Culture media were harvested at 96 h.p.i. to detect viral replication (P1, P2, and P4, n=3; P3, n=2). Data represent mean and SD of the indicated number (n) of biological replicates from a representative experiment. Statistical significance (in P1, P2, and P4) was determined using a two-tailed Student's t-test. *P < 0.05, **P < 0.01. *ns* not significant. Source data are provided as a Source Data file.

Supplementary Figure 5. Orthogonal views of human nasal organoid with HRV-C infection.

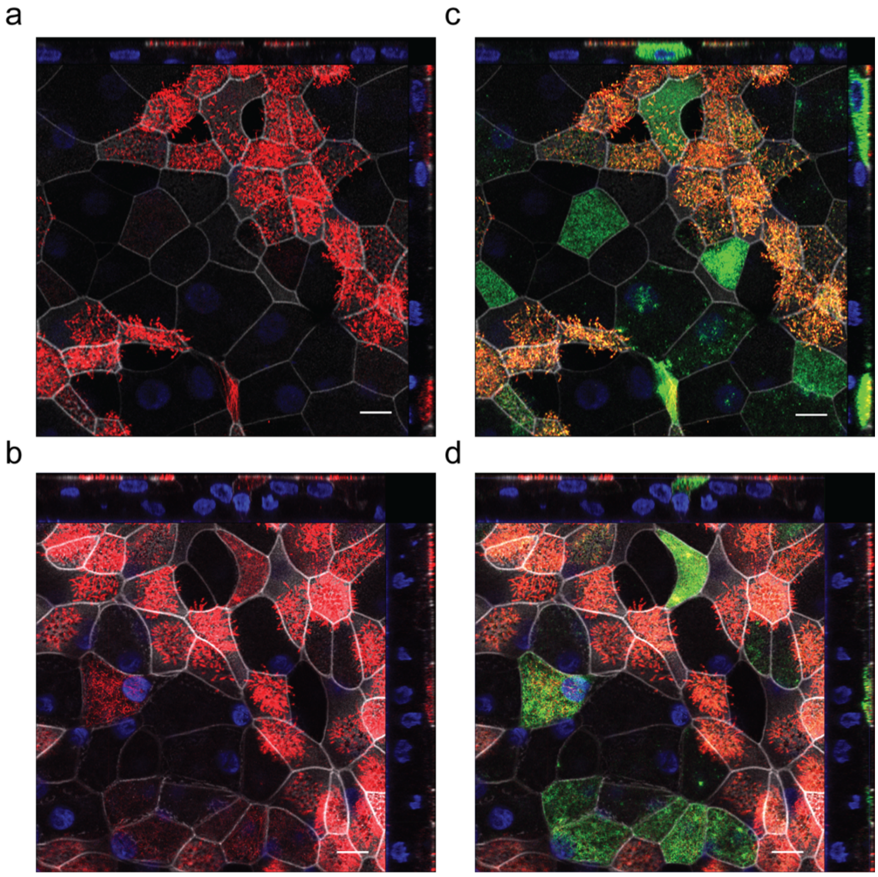

(a-d) Orthogonal views of human nasal organoid with HRV-C infection. Nasal organoids were fixed and double-stained with an α-VP3 (green) and α-ACCTUB (red). Orthogonal views of confocal images with en face and cross-section are shown. Nuclei and actin filaments were counterstained with DAPI (blue) and Phalloidin-647 (white), respectively. (a, b) Three channels are shown: α-ACCTUB (red), DAPI (blue), and Phalloidin-647 (white). (c, d) Four channels are shown: α-VP3 (green), α-ACCTUB (red), DAPI (blue), and Phalloidin-647 (white). The experiment was repeated three times independently with similar results. Scale bar, 10 μm.

Supplementary Figure 6. Host transcriptional response in human airway and nasal organoids.

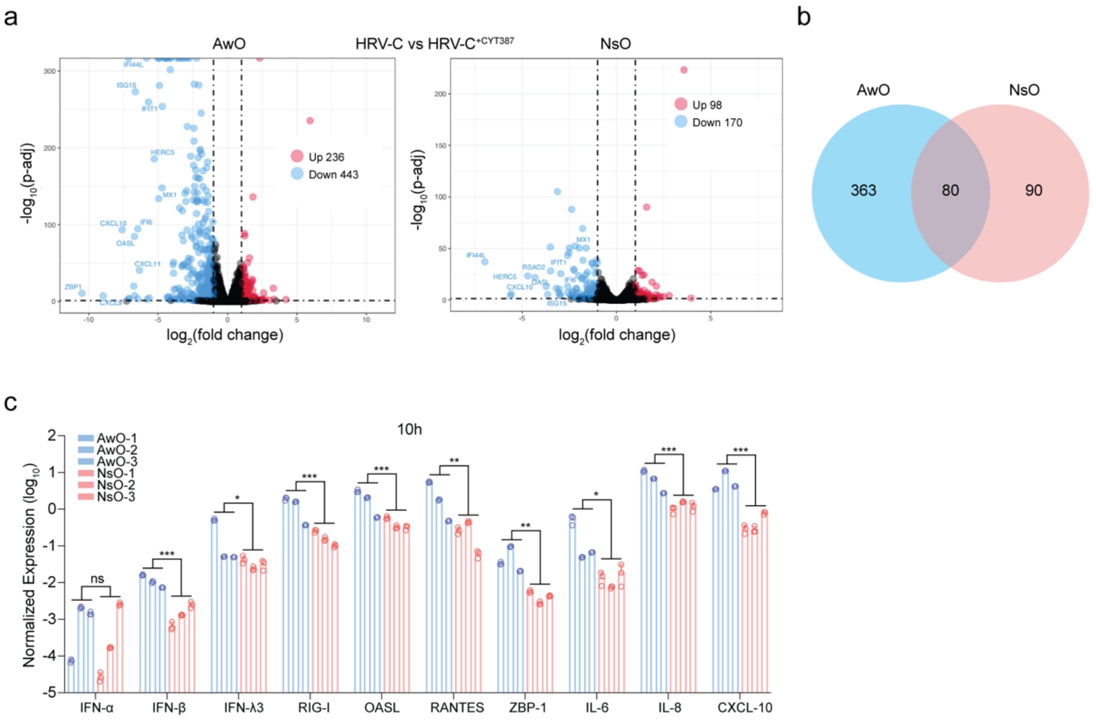

HRV-C3 infected airway (AwO) organoids and nasal organoids (NsO) treated with CYT387 (HRV-C$^{+CYT387}$) or DMSO (HRV-C) were applied to RNA sequencing analysis. (a) Volcano plot shows DEGs in the HRV-C-infected airway and nasal organoids with CYT387 treatment compared with HRV-C-infected organoids. DEGs with a log2(fold change) >1 and <-1 are shown in red and blue respectively. (b) Venn diagram shows the numbers of unique and common downregulated DEGs in the infected airway and nasal organoids. (c) Three lines of airway and nasal organoids were treated with 10 µg/ml Poly(I:C) or mock-treated. At 10 h post-treatment, organoids were harvested for RT-qPCR (n=3). Results showed the GAPDH normalized expression level of innate immune molecules in the airway and nasal organoids. Data represent mean and SD of the indicated number (n) of biological replicates from a representative experiment. Statistical significance (in c) was determined using a two-tailed Student's t-test. *P < 0.05, **P < 0.01, ***P < 0.001. *ns* not significant. Source data are provided as a Source Data file for Supplementary Figure 6c.

Supplementary Figure 7. High CDHR3 expression on the apical surface of the airway organoids.

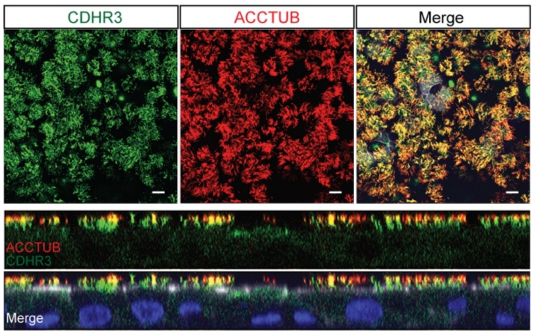

Airway organoids were fixed and double-stained with an α-CDHR3 (green) and α-ACCTUB (red). Confocal images of en face (top) and cross-section (bottom) are shown. Nuclei and actin filaments were counterstained with DAPI (blue) and Phalloidin-647 (white), respectively. The experiment was performed three times independently with similar results. Scale bar, 10 µm.

Supplementary Table 1. Antibody list.

Name	Supplier	Cat. no.	Lot no.	Dilution (IF)	Dilution (Flow)
Mouse IgG	abcam	ab91353	GR3327311-3	1:100	1:100
Rabbit IgG	abcam	ab172730	GR3284310-8	1:100	1:100
Rhinovirus VP3	Invitrogen	MA5-18249	YD3903932	1:100	1:100
CDHR3	abcam	ab121337	1016309-2	1:100	1:100
CDHR3	Sigma-Aldrich	HPA011218	000006575	1:100	-
TUBULIN	abcam	ab179509	GR252919-6	1:100	1:1000
TUBULIN	Sigma-Aldrich	T7941	088M4793	1:100	-
Goat anti-Mouse 488	invitrogen	A-11001	2090562	1:200	1:300
Goat anti-Mouse 594	invitrogen	A-11005	1750828	1:200	-
Goat anti-Rabbit 488	invitrogen	A-11034	1885241	1:200	1:300
Goat anti-Rabbit 594	invitrogen	A-11037	1608397	1:200	1:300

Supplementary Table 2. Primer sequences for qPCR analysis

Gene	Sequence
IFN-α	F 5'-AGAATCACTCTCTATCTGAAAGAGAAGAAATA-3'<br>R 5'-TCATGATTTCTGCTCTGACAACCT-3'
IFN-β	F 5'-CTTGGATTCCTACAAAGAAGCAGC-3'<br>R 5'-TCCTCCTTCTGGAACTGCTGCA-3'
IFN-λ3	F 5'-TCGAGGCCTCTGTCACCTTC-3'<br>R 5'-AGGTTGCATGACTGGCGGAA-3'
RIG-I	F 5'-AGAGCACTTGTGGACGCTTT-3'<br>R 5'-GCCACGTCCAGTCAATATGC-3'
OASL	F 5'-AAACCATGTGGCAAAGCCAG-3'<br>R 5'-GCTCTGTAGGCAGGCACAAT-3'
RANTES	F 5'-TCATTGCTACTGCCCTCTGC-3'<br>R 5'-TACTCCTTGATGTGGGCACG-3'
ZBP1	F 5'-GCAAACTCCGAAGCCATCCAGA-3'<br>R 5'-CCAAGTTGAGGAATCACCTGGTG-3'
IL-6	F 5'-AGACAGCCACTCACCTCTTCAG-3'<br>R 5'-TTCTGCCAGTGCCTCTTTGCTG-3'
IL-8	F 5'-GAGAGTGATTGAGAGTGGACCAC-3'<br>R 5'-CACAACCCTCTGCACCCAGTTT-3'
IP-10	F 5'-GAAATTATTCCTGCAAGCCAATTT-3'<br>R 5'-TCACCCTTCTTTTTCATTGTAGCA-3'
HRV-C 5' UTR	F1 5'-AGCCYGCGTGGCKGCC-3'<br>F2 5'-AGCCYGCGTGGTGCCC-3'<br>R 5'-GAAACACGGACACCCAAAGTAGT-3'<br>Probe 5' HEX-TCCGGCCCCTGAATGYGGCTAA-lABkFQ 3'

Supplementary Information File. The whole genome sequence of the passaged HRV-C.

```
HRV-C_P0 1      NNNNNNNNNNNNNNNNNNNNNNNNNNNNNNNNNNNNNNNNNNNNNNNNNNNNNNNNNNNNNNNNNNNNNNNNNNNNNNNN
HRV-C_P1 1      TTAAAACTGAATCATGGTTGTTCCCACCATGATTACCCACGCGGTGCAGTGGTCTTGTATCACGGTACATTTCCATACCA
HRV-C_P6 1      TTAAAACTGAATCATGGTTGTTCCCACCATGATTACCCACGCGGTGCAGTGGTCTTGTATCACGGTACATTTCCATACCA

HRV-C_P0 81     NNNNNNNNNNNNNNNNNNNNNNNNNNNNNNNNNNNNNNNNNNNNNNNNNNNNNNNNNNNNNNNNNNNNNNNNNNNNNNNN
HRV-C_P1 81     GTTTTATACACCCCACCCCGAAACTCATAGAAGTTTGTACACAATGACCAATAGGCGGTGGCCATCCAGGTCACTAATGG
HRV-C_P6 81     GTTTTATACACCCCACCCCGAAACTCATAGAAGTTTGTACACAATGACCAATAGGCGGTGGCCATCCAGGTCACTAATGG

HRV-C_P0 161    NCAAGCACTTCTGTTTCCCCGGCACCCTTGTATACGCTTCACCCGAGGCGAAAAACGAGGTTGTCGTTATCCGCAAAGTG
HRV-C_P1 161    TCAAGCACTTCTGTTTCCCCGGCACCCTTGTATACGCTTCACCCGAGGCGAAAAACGAGGTTGTCGTTATCCGCAAAGTG
HRV-C_P6 161    TCAAGCACTTCTGTTTCCCCGGCACCCTTGTATACGCTTCACCCGAGGCGAAAAACGAGGTTGTCGTTATCCGCAAAGTG

HRV-C_P0 241    CCTACGAAAAGCCTAGTAACACTTTGAAAACCTATGGTTGGTCGCTCAGCTGTTCACCCAACAGTAGACCTGGCAGATGA
HRV-C_P1 241    CCTACGAAAAGCCTAGTAACACTTTGAAAACCTATGGTTGGTCGCTCAGCTGTTCACCCAACAGTAGACCTGGCAGATGA
HRV-C_P6 241    CCTACGAAAAGCCTAGTAACACTTTGAAAACCTATGGTTGGTCGCTCAGCTGTTCACCCAACAGTAGACCTGGCAGATGA

HRV-C_P0 321    GGCTAGACACTCCCCACCAGCGATGGTGGTCTAGCCTGCGTGGCTGCCTGCACACCCCACCGGGTGTGAAGCCAGAAAGT
HRV-C_P1 321    GGCTAGACACTCCCCACCAGCGATGGTGGTCTAGCCTGCGTGGCTGCCTGCACACCCCACCGGGTGTGAAGCCAGAAAGT
HRV-C_P6 321    GGCTAGACACTCCCCACCAGCGATGGTGGTCTAGCCTGCGTGGCTGCCTGCACACCCCACCGGGTGTGAAGCCAGAAAGT

HRV-C_P0 401    GGACAAGGTGTGAAGAGCCTATTGTGCTCACTTTGAGTCCTCCGGCCCCTGAATGTGGCTAACCCTAACCCCGTAGCTGT
HRV-C_P1 401    GGACAAGGTGTGAAGAGCCTATTGTGCTCACTTTGAGTCCTCCGGCCCCTGAATGTGGCTAACCCTAACCCCGTAGCTGT
HRV-C_P6 401    GGACAAGGTGTGAAGAGCCTATTGTGCTCACTTTGAGTCCTCCGGCCCCTGAATGTGGCTAACCCTAACCCCGTAGCTGT

HRV-C_P0 481    TGCATGTAATCCAACATGTATGCAGTCGTAATGGGCAACTATGGGATGGGACCAACTACTTTGGGTGTCCGTGTTTCCTG
HRV-C_P1 481    TGCATGTAATCCAACATGTATGCAGTCGTAATGGGCAACTATGGGATGGGACCAACTACTTTGGGTGTCCGTGTTTCCTG
HRV-C_P6 481    TGCATGTAATCCAACATGTATGCAGTCGTAATGGGCAACTATGGGATGGGACCAACTACTTTGGGTGTCCGTGTTTCCTG

HRV-C_P0 561    TTTTACTTTTTCATTGCTTATGGTGACAATTGTATCTGATACACTTGTTACCATGGGCGCTCAAGTGTCTAGACAGAGTG
HRV-C_P1 561    TTTTACTTTTTCATTGCTTATGGTGACAATTGTATCTGATACACTTGTTACCATGGGCGCTCAAGTGTCTAGACAGAGTG
HRV-C_P6 561    TTTTACTTTTTCATTGCTTATGGTGACAATTGTATCTGATACACTTGTTACCATGGGCGCTCAAGTGTCTAGACAGAGTG

HRV-C_P0 641    TTGGAAGCCATGAAACAATGATCCACGCTGGGACTGGGGCTGTGGTCAAATACTTTAATGTCAACTACTACAAAGATGCA
HRV-C_P1 641    TTGGAAGCCATGAAACAATGATCCACGCTGGGACTGGGGCTGTGGTCAAATACTTTAATGTCAACTACTACAAAGATGCA
HRV-C_P6 641    TTGGAAGCCATGAAACAATGATCCACGCTGGGACTGGGGCTGTGGTCAAATACTTTAATGTCAACTACTACAAAGATGCA

HRV-C_P0 721    GCAAGTTCTGGATTGACCAAGCAAGACTTCTCTCAAGATCCATCTAAATTCACTCAACCTGTGGCAGATATCTTGACAAA
HRV-C_P1 721    GCAAGTTCTGGATTGACCAAGCAAGACTTCTCTCAAGATCCATCTAAATTCACTCAACCTGTGGCAGATATCTTGACAAA
HRV-C_P6 721    GCAAGTTCTGGATTGACCAAGCAAGACTTCTCTCAAGATCCATCTAAATTCACTCAACCTGTGGCAGATATCTTGACAAA

HRV-C_P0 801    CCCTGCATTGATGTCTCCTTCCATTGAAGCATGTGGGTTTTCCGATAGGCTCAAGCAAATCACTATTGGGAACTCCACTA
HRV-C_P1 801    CCCTGCATTGATGTCTCCTTCCATTGAAGCATGTGGGTTTTCCGATAGGCTCAAGCAAATCACTATTGGGAACTCCACTA
HRV-C_P6 801    CCCTGCATTGATGTCTCCTTCCATTGAAGCATGTGGGTTTTCCGATAGGCTCAAGCAAATCACTATTGGGAACTCCACTA

HRV-C_P0 881    TCACAACACAAGATGCTGTTAACACAATCGTTGCATATGGTGAATGGCCTAGTTACTTATCAGACATAGATGCAACCTCA
HRV-C_P1 881    TCACAACACAAGATGCTGTTAACACAATCGTTGCATATGGTGAATGGCCTAGTTACTTATCAGACATAGATGCAACCTCA
HRV-C_P6 881    TCACAACACAAGATGCTGTTAACACAATCGTTGCATATGGTGAATGGCCTAGTTACTTATCAGACATAGATGCAACCTCA

HRV-C_P0 961    GTTGACAAACCCACCCATCCTGAAACATCGTCAGACAGGTTCTACACGCTGAAGAGCGTTACTTGGGAGGTTGGATCATC
HRV-C_P1 961    GTTGACAAACCCACCCATCCTGAAACATCGTCAGACAGGTTCTACACGCTGAAGAGCGTTACTTGGGAGGTTGGATCATC
HRV-C_P6 961    GTTGACAAACCCACCCATCCTGAAACATCGTCAGACAGGTTCTACACGCTGAAGAGCGTTACTTGGGAGGTTGGATCATC

HRV-C_P0 1041   AGGCTGGTGGTGGAAATTACCAGACTGTTTAAGGGACATGGGTGTATTTGGACAAAACATGTACCACCATGCTATGGGAA
HRV-C_P1 1041   AGGCTGGTGGTGGAAATTACCAGACTGTTTAAGGGACATGGGTGTATTTGGACAAAACATGTACCACCATGCTATGGGAA
HRV-C_P6 1041   AGGCTGGTGGTGGAAATTACCAGACTGTTTAAGGGACATGGGTGTATTTGGACAAAACATGTACCACCATGCTATGGGAA

HRV-C_P0 1121   GATCTGGGTATATTATACACACTCAGTGTAATGCCACCAAATTTCATAGCGGGTGCTTACTAGTAGCAGTAGTGCCTGAA
HRV-C_P1 1121   GATCTGGGTATATTATACACACTCAGTGTAATGCCACCAAATTTCATAGCGGGTGCTTACTAGTAGCAGTAGTGCCTGAA
HRV-C_P6 1121   GATCTGGGTATATTATACACACTCAGTGTAATGCCACCAAATTTCATAGCGGGTGCTTACTAGTAGCAGTAGTGCCTGAA
```

```
HRV-C_P0 1201 CATCAGTTAGCATACATTGGAGGTGACAACACCCGAGTCAAATATAAGCACACACACCCAGGTGAATTAGGCCATAAAAT
HRV-C_P1 1201 CATCAGTTAGCATACATTGGAGGTGACAACACCCGAGTCAAATATAAGCACACACACCCAGGTGAATTAGGCCATAAAAT
HRV-C_P6 1201 CATCAGTTAGCATACATTGGAGGTGACAACACCCGAGTCAAATATAAGCACACACACCCAGGTGAATTAGGCCATAAAAT

HRV-C_P0 1281 TGGTAGTAACTCAGAAAGAGGTGATAATCAACCAGATGAAAACCCTTTCTTCAACTGCAATGGAACTTTGCTGGGAAATT
HRV-C_P1 1281 TGGTAGTAACTCAGAAAGAGGTGATAATCAACCAGATGAAAACCCTTTCTTCAACTGCAATGGAACTTTGCTGGGAAATT
HRV-C_P6 1281 TGGTAGTAACTCAGAAAGAGGTGATAATCAACCAGATGAAAACCCTTTCTTCAACTGCAATGGAACTTTGCTGGGAAATT

HRV-C_P0 1361 TAACAATCTTCCCACACCAACTCATAAACTTGAGGACAAACAACTCAAGCACCATTGTTGTGCCCTATATTAACTGCACA
HRV-C_P1 1361 TAACAATCTTCCCACACCAACTCATAAACTTGAGGACAAACAACTCAAGCACCATTGTTGTGCCCTATATTAACTGCACA
HRV-C_P6 1361 TAACAATCTTCCCACACCAACTCATAAACTTGAGGACAAACAACTCAAGCACCATTGTTGTGCCCTATATTAACTGCACA

HRV-C_P0 1441 CCCATGGACAGCATGTTACGCCATAATAATGTGTCACTTGTTATTATACCTATATGTCCTCTGCGAGCACCAGCTACAGC
HRV-C_P1 1441 CCCATGGACAGCATGTTACGCCATAATAATGTGTCACTTGTTATTATACCTATATGTCCTCTGCGAGCACCAGCTACAGC
HRV-C_P6 1441 CCCATGGACAGCATGTTACGCCATAATAATGTGTCACTTGTTATTATACCTATATGTCCTCTGCGAGCACCAGCTACAGC

HRV-C_P0 1521 TCCCTCAACCTTGCCAATCACAATATCCATTGCCCCCATCAAATCAGAATTTTCGGGCGCAAGACAGTCTGCTAAAGCAC
HRV-C_P1 1521 TCCCTCAACCTTGCCAATCACAATATCCATTGCCCCCATCAAATCAGAATTTTCGGGCGCAAGACAGTCTGCTAAAGCAC
HRV-C_P6 1521 TCCCTCAACCTTGCCAATCACAATATCCATTGCCCCCATCAAATCAGAATTTTCGGGCGCAAGACAGTCTGCTAAAGCAC

HRV-C_P0 1601 AAGGCCTACCAGTGAGAATGCCAAGTGGAGCCAACCAGTTCATGACAACAGAGGATGAACAGTCTCCAAACATACTCCCC
HRV-C_P1 1601 AAGGCCTACCAGTGAGAATGCCAAGTGGAGCCAACCAGTTCATGACAACAGAGGATGAACAGTCTCCAAACATACTCCCC
HRV-C_P6 1601 AAGGCCTACCAGTGAGAATGCCAAGTGGAGCCAACCAGTTCATGACAACAGAGGATGAACAGTCTCCAAACATACTCCCC

HRV-C_P0 1681 AGCTTCCATTCAACTAAACAAATTCACATACCTGGTGAAATTAAGACAATTATGGACATGGCTCGCGTAGACTCCTTTAT
HRV-C_P1 1681 AGCTTCCATTCAACTAAACAAATTCACATACCTGGTGAAATTAAGACAATTATGGACATGGCTCGCGTAGACTCCTTTAT
HRV-C_P6 1681 AGCTTCCATTCAACTAAACAAATTCACATACCTGGTGAAATTAAGACAATTATGGACATGGCTCGCGTAGACTCCTTTAT

HRV-C_P0 1761 GCCTATAAATAATGTGAATCAGTCTCATACACTGGGGGTTTACTACATCACAATACCAAATGTGAGCAGTGAGAGGAAAA
HRV-C_P1 1761 GCCTATAAATAATGTGAATCAGTCTCATACACTGGGGGTTTACTACATCACAATACCAAATGTGAGCAGTGAGAGGAAAA
HRV-C_P6 1761 GCCTATAAATAATGTGAATCAGTCTCATACACTGGGGGTTTACTACATCACAATACCAAATGTGAGCAGTGAGAGGAAAA

HRV-C_P0 1841 TACTGGCTATACCACTAGATATGAGTAATACACTTTTCTCTACAACACTTATGGGGGAAGTTCTAAACTACTACTCAAAT
HRV-C_P1 1841 TACTGGCTATACCACTAGATATGAGTAATACACTTTTCTCTACAACACTTATGGGGGAAGTTCTAAACTACTACTCAAAT
HRV-C_P6 1841 TACTGGCTATACCACTAGATATGAGTAATACACTTTTCTCTACAACACTTATGGGGGAAGTTCTAAACTACTACTCAAAT

HRV-C_P0 1921 TGGTCTGGGTCAATCAAACTTACATTCTTCTGTGTTTGTGACAGCTTCAGCACTGGTAAGTTCTTGATAGCATACACCCC
HRV-C_P1 1921 TGGTCTGGGTCAATCAAACTTACATTCTTCTGTGTTTGTGACAGCTTCAGCACTGGTAAGTTCTTGATAGCATACACCCC
HRV-C_P6 1921 TGGTCTGGGTCAATCAAACTTACATTCTTCTGTGTTTGTGACAGCTTCAGCACTGGTAAGTTCTTGATAGCATACACCCC

HRV-C_P0 2001 TCCAGGTGCTGACATCCCAGAGACACGCCGTGATGCAATGCTCGGCACACATGTCGTGTGGGACCTGGGTCTCCAATCAT
HRV-C_P1 2001 TCCAGGTGCTGACATCCCAGAGACACGCCGTGATGCAATGCTCGGCACACATGTCGTGTGGGACCTGGGTCTCCAATCAT
HRV-C_P6 2001 TCCAGGTGCTGACATCCCAGAGACACGCCGTGATGCAATGCTCGGCACACATGTCGTGTGGGACCTGGGTCTCCAATCAT

HRV-C_P0 2081 CATGCCACCTTGTTGTACCTTGGATCAGCTCTCATTTCTTCAGGAGGACCAGAAAGGACAACTACACAGAGAGTGGGTTC
HRV-C_P1 2081 CATGCCACCTTGTTGTACCTTGGATCAGCTCTCATTTCTTCAGGAGGACCAGAAAGGACAACTACACAGAGAGTGGGTTC
HRV-C_P6 2081 CATGCCACCTTGTTGTACCTTGGATCAGCTCTCATTTCTTCAGGAGGACCAGAAAGGACAACTACACAGAGAGTGGGTTC

HRV-C_P0 2161 GTTACATTGTGGCATCAGACAGCATTTGTGTCCAATGGAACTTCAGGATCCATAATGGTCACCTGCTCTGCTTGCTCTGA
HRV-C_P1 2161 GTTACATTGTGGCATCAGACAGCATTTGTGTCCAATGGAACTTCAGGATCCATAATGGTCACCTGCTCTGCTTGCTCTGA
HRV-C_P6 2161 GTTACATTGTGGCATCAGACAGCATTTGTGTCCAATGGAACTTCAGGATCCATAATGGTCACCTGCTCTGCTTGCTCTGA

HRV-C_P0 2241 CATGTCAGTCCGCATGTTGCGAGACACACCCATGATGAAGCAAGAAGATAACATCACTCAAAACCCAGTGGAAGAGTTTG
HRV-C_P1 2241 CATGTCAGTCCGCATGTTGCGAGACACACCCATGATGAAGCAAGAAGATAACATCACTCAAAACCCAGTGGAAGAGTTTG
HRV-C_P6 2241 CATGTCAGTCCGCATGTTGCGAGACACACCCATGATGAAGCAAGAAGATAACATCACTCAAAACCCAGTGGAAGAGTTTG

HRV-C_P0 2321 TAGAGCACACATTAAAAGAGGTGTTGGTAGTTCCAGACACTCAAGCTTCAGGACCTGTTCATACCACCAAACCACAAGCT
HRV-C_P1 2321 TAGAGCACACATTAAAAGAGGTGTTGGTAGTTCCAGACACTCAAGCTTCAGGACCTGTTCATACCACCAAACCACAAGCT
HRV-C_P6 2321 TAGAGCACACATTAAATGAGGTGTTGGTAGTTCCAGACACTCAAGCTTCAGGACCTGTTCATACCACCAAACCACAAGCT
```

```
HRV-C_P0 2401 CTAGGTGCTGTAGAAATAGGAGCAACTGCTGATGTGGGCCCAGAAACATTAATTGAGACAAGATACGTAATGAATGACAA
HRV-C_P1 2401 CTAGGTGCTGTAGAAATAGGAGCAACTGCTGATGTGGGCCCAGAAACATTAATTGAGACAAGATACGTAATGAATGACAA
HRV-C_P6 2401 CTAGGTGCTGTAGAAATAGGAGCAACTGCTGATGTGGGCCCAGAAACATTAATTGAGACAAGATACGTAATGAATGACAA

HRV-C_P0 2481 CACAAATGCTGAGGCAGCAGTGGAGAACTTCCTGGGAAGATCTGCTTTGTGGGCAAATCTAAGGCTGGATCAGGGTTTCA
HRV-C_P1 2481 CACAAATGCTGAGGCAGCAGTGGAGAACTTCCTGGGAAGATCTGCTTTGTGGGCAAATCTAAGGCTGGATCAGGGTTTCA
HRV-C_P6 2481 CACAAATGCTGAGGCAGCAGTGGAGAACTTCCTGGGAAGATCTGCTTTGTGGGCAAATCTAAGGCTGGATCAGGGTTTCA

HRV-C_P0 2561 GGAAATGGGAGATAAACTTCCAAGAACATGCCCAAGTCAGGAAAAAGTTTGAGATGTTCACATATGTTAGATTTGACCTG
HRV-C_P1 2561 GGAAATGGGAGATAAACTTCCAAGAACATGCCCAAGTCAGGAAAAAGTTTGAGATGTTCACATATGTTAGATTTGACCTG
HRV-C_P6 2561 GGAAATGGGAGATAAACTTCCAAGAACATGCCCAAGTCAGGAAAAAGTTTGAGATGTTCACATATGTTAGATTTGACCTG

HRV-C_P0 2641 GAAATAACAATTGTCACCAACAATAAGGGATTGATGCAAATTATGTTTGTACCTCCAGGAATCACACCTCCAGGAGGGAA
HRV-C_P1 2641 GAAATAACAATTGTCACCAACAATAAGGGATTGATGCAAATTATGTTTGTACCTCCAGGAATCACACCTCCAGGAGGGAA
HRV-C_P6 2641 GAAATAACAATTGTCACCAACAATAAGGGATTGATGCAAATTATGTTTGTACCTCCAGGAATCACACCTCCAGGAGGGAA

HRV-C_P0 2721 AGATGGTAGGGAATGGGACACAGCTTCTAACCCTAGTGTTTTCTTCCAACCAAACTCTGGTTTCCCGAGATTCACGATAC
HRV-C_P1 2721 AGATGGTAGGGAATGGGACACAGCTTCTAACCCTAGTGTTTTCTTCCAACCAAACTCTGGTTTCCCGAGATTCACGATAC
HRV-C_P6 2721 AGATGGTAGGGAATGGGACACAGCTTCTAACCCTAGTGTTTTCTTCCAACCAAACTCTGGTTTCCCGAGATTCACGATAC

HRV-C_P0 2801 CCTTCACAGGGTTGGGATCGGCATACTATATGTTCTATGATGGATATGATGGTACTGACGATGCCAATATAAATTATGGA
HRV-C_P1 2801 CCTTCACAGGGTTGGGATCGGCATACTATATGTTCTATGATGGATATGATGGTACTGACGATGCCAATATAAATTATGGA
HRV-C_P6 2801 CCTTCACAGGGTTGGGATCGGCATACTATATGTTCTATGATGGATATGATGGTACTGACGATGCCAATACAAATTATGGA

HRV-C_P0 2881 ATCTCATTAACAAATGACATGGGAACTTTATGTTTCCGTGCTTTGGATGGAACTGGAGCAAGTGATATCAAAGTGTTTGG
HRV-C_P1 2881 ATCTCATTAACAAATGACATGGGAACTTTATGTTTCCGTGCTTTGGATGGAACTGGAGCAAGTGATATCAAAGTGTTTGG
HRV-C_P6 2881 ATCTCATTAACAAATGACATGGGAACTTTATGTTTCCGTGCTTTGGATGGAACTGGAGCAAGTGATATCAAAGTGTTTGG

HRV-C_P0 2961 CAAACCCAAGCACATAACTGCATGGATTCCCCGCCCACCCAGAGCAACCCAATACCTGCACAAATTTAGCACCAACTATA
HRV-C_P1 2961 CAAACCCAAGCACATAACTGCATGGATTCCCCGCCCACCCAGAGCAACCCAATACCTGCACAAATTTAGCACCAACTATA
HRV-C_P6 2961 CAAACCCAAGCACATAACTGCATGGATTCCCCGCCCACCCAGAGCAACCCAATACCTGCACAAATTTAGCACCAACTATA

HRV-C_P0 3041 ACAAGCCAAAGACAACTGGCAGCACTGAGTTGGAACCAAAACACTTCTTTAAATACAGACAAGACATTACAAGTATTACC
HRV-C_P1 3041 ACAAGCCAAAGACAACTGGCAGCACTGAGTTGGAACCAAAACACTTCTTTAAATACAGACAAGACATTACAAGTATTACC
HRV-C_P6 3041 ACAAGCCAAAGACAACTGGCAGCACTGAGTTGGAACCAAAACACTTCTTTAAATACAGACAAGACATTACAAGTATTACC

HRV-C_P0 3121 AACCTGGGACCCAGTGACATGTATGTTCACACAAAAGAAGCCATCTATAAGTGTGCACACCTAACTGAACCTAATGAAAA
HRV-C_P1 3121 AACCTGGGACCCAGTGACATGTATGTTCACACAAAAGAAGCCATCTATAAGTGTGCACACCTAACTGAACCTAATGAAAA
HRV-C_P6 3121 AACCTGGGACCCAGTGACATGTATGTTCACACAAAAGAAGCCATCTATAAGTGTGCACACCTAACTGAACCTAATGAAAA

HRV-C_P0 3201 CACCATACTCCTGGCTATATCATCAGATCTTCAAGTTGACAGCACAAGCATCCCTGGCCCTGACAATATACCTACCTGTG
HRV-C_P1 3201 CACCATACTCCTGGCTATATCATCAGATCTTCAAGTTGACAGCACAAGCATCCCTGGCCCTGACAATATACCTACCTGTG
HRV-C_P6 3201 CACCATACTCCTGGCTATATCATCAGATCTTCAAGTTGACAGCACAAGCATCCCTGGCCCTGACAATATACCTACCTGTG

HRV-C_P0 3281 ATTGCACCACAGGTACCTATTATTGCAAAAGCAAGGACAGATACTATCCAGTGAACCTAACACACCACAGTTGGTATGTA
HRV-C_P1 3281 ATTGCACCACAGGTACCTATTATTGCAAAAGCAAGGACAGATACTATCCAGTGAACCTAACACACCACAGTTGGTATGTA
HRV-C_P6 3281 ATTGCACCACAGGTACCTATTATTGCAAAAGCAAGGACAGATACTATCCAGTGAACCTAACACACCACAGTTGGTATGTA

HRV-C_P0 3361 ATACAAGAATCCAGCTACTACCCAAAACACATTCAGTATGATATACTGATTGGCGAAGGACCTTGCTCTCCAGGGGATTG
HRV-C_P1 3361 ATACAAGAATCCAGCTACTACCCAAAACACATTCAGTATGATATACTGATTGGCGAAGGACCTTGCTCTCCAGGGGATTG
HRV-C_P6 3361 ATACAAGAATCCAGCTACTACCCAAAACACATTCAGTATGATATACTGATTGGCGAAGGACCTTGCTCTCCAGGGGATTG

HRV-C_P0 3441 TGGTGGTAAACTACTGTGTATACATGGAACCATAGGGATGGTGACAGCAGGTGGAGAGAATCATGTTGCATTCATAGACT
HRV-C_P1 3441 TGGTGGTAAACTACTGTGTATACATGGAACCATAGGGATGGTGACAGCAGGTGGAGAGAATCATGTTGCATTCATAGACT
HRV-C_P6 3441 TGGTGGTAAACTACTGTGTATACATGGAACCATAGGGATGGTGACAGCAGGTGGAGAGAATCATGTTGCATTCATAGACT

HRV-C_P0 3521 TGAGAAACTACAGCTCACTTAGTGAACATCAGGGTATCACAGACTATGTCACACAACTGGGAAATGCATTTGGAGATGGG
HRV-C_P1 3521 TGAGAAACTACAGCTCACTTAGTGAACATCAGGGTATCACAGACTATGTCACACAACTGGGAAATGCATTTGGAGATGGG
HRV-C_P6 3521 TGAGAAACTACAGCTCACTTAGTGAACATCAGGGTATCACAGACTATGTCACACAACTGGGAAATGCATTTGGAGATGGG
```

```
HRV-C_P0 3601 TTCACAAGTTCTATTAGGGAAACACTAGTATCTGCATGTGATGTGTCAGACAAACTCACTGGAAAAATCATCAAATGGAC
HRV-C_P1 3601 TTCACAAGTTCTATTAGGGAAACACTAGTATCTGCATGTGATGTGTCAGACAAACTCACTGGAAAAATCATCAAATGGAC
HRV-C_P6 3601 TTCACAAGTTCTATTAGGGAAACACTAGTATCTGCATGTGATGTGTCAGACAAACTCACTGGAAAAATCATCAAATGGAC

HRV-C_P0 3681 GATTAGACTGATTAGTGCCTTATCAATTATAGTTAGAAACAGCACTGACACCCCCACTGTCATAGCCACACTTGCCCTGT
HRV-C_P1 3681 GATTAGACTGATTAGTGCCTTATCAATTATAGTTAGAAACAGCACTGACACCCCCACTGTCATAGCCACACTTGCCCTGT
HRV-C_P6 3681 GATTAGACTGATTAGTGCCTTATCAATTATAGTTAGAAACAGCACTGACACCCCCACTGTCATAGCCACACTTGCCCTGT

HRV-C_P0 3761 TAGGATGCTCCGGTTCACCATGGCGCTACTTGAAAGACAAAGTTTGCAAGTGGCTGGGTATCGATCCACCACCTAGTAGA
HRV-C_P1 3761 TAGGATGCTCCGGTTCACCATGGCGCTACTTGAAAGACAAAGTTTGCAAGTGGCTGGGTATCGATCCACCACCTAGTAGA
HRV-C_P6 3761 TAGGATGCTCCGGTTCACCATGGCGCTACTTGAAAGACAAAGTTTGCAAGTGGCTGGGTATCGATCCACCACCTAGTAGA

HRV-C_P0 3841 CAGAGTGACTCATGGCTAAAAAAATTCACCGAGTTCTGTAATGCAGCCCGTGGGCTAGAATGGATCGGTGATAAATTATC
HRV-C_P1 3841 CAGAGTGACTCATGGCTAAAAAAATTCACCGAGTTCTGTAATGCAGCCCGTGGGCTAGAATGGATCGGTGATAAATTATC
HRV-C_P6 3841 CAGAGTGACTCATGGCTAAAAAAATTCACCGAGTTCTGTAATGCAGCCCGTGGGCTAGAATGGATCGGTGATAAATTATC

HRV-C_P0 3921 TAAATTCATTGATTGGCTGAAAGGAAAAATACTACCCACTCTCCGACGCAAGTCAGATACACTTAAGGAGTGCAAAAAGA
HRV-C_P1 3921 TAAATTCATTGATTGGCTGAAAGGAAAAATACTACCCACTCTCCGACGCAAGTCAGATACACTTAAGGAGTGCAAAAAGA
HRV-C_P6 3921 TAAATTCATTGATTGGCTGAAAGGAAAAATACTACCCACTCTCCGACGCAAGTCAGATACACTTAAGGAGTGCAAAAAGA

HRV-C_P0 4001 TTCCATTGTACAAAGAACAAGTCAAAGCATTTGCCACAGCATCAGAGGAAGCACAAGATGAATTAATGGTAAAGGTCAAC
HRV-C_P1 4001 TTCCATTGTACAAAGAACAAGTCAAAGCATTTGCCACAGCATCAGAGGAAGCACAAGATGAATTAATGGTAAAGGTCAAC
HRV-C_P6 4001 TTCCATTGTACAAAGAACAAGTCAAAGCATTTGCCACAGCATCAGAGGAAGCACAAGATGAATTAATGGTAAAGGTCAAC

HRV-C_P0 4081 AAACTTGAAAAAGGGCTCCAGGATCTTGCCCCATTATACGCACTAGAAGCCAAACAAGTGCGCGAGATGTCTAGGGAACT
HRV-C_P1 4081 AAACTTGAAAAAGGGCTCCAGGATCTTGCCCCATTATACGCACTAGAAGCCAAACAAGTGCGCGAGATGTCTAGGGAACT
HRV-C_P6 4081 AAACTTGAAAAAGGGCTCCAGGATCTTGCCCCATTATACGCACTAGAAGCCAAACAAGTGCGCGAGATGTCTAGGGAACT

HRV-C_P0 4161 ACAGCGCATGATGTGCTACAAGAAAACCCACAGAACAGAACCTGTCTGCATATTATTGCATGGACAACCAGGATGTGGTA
HRV-C_P1 4161 ACAGCGCATGATGTGCTACAAGAAAACCCACAGAACAGAACCTGTCTGCATATTATTGCATGGACAACCAGGATGTGGTA
HRV-C_P6 4161 ACAGCGCATGATGTGCTACAAGAAAACCCACAGAACAGAACCTGTCTGCATATTATTGCATGGACAACCAGGATGTGGTA

HRV-C_P0 4241 AGTCCTTGGCCTCTAAAGTTATCGCTAGAGGCCTCACTAGTGAGAGCAATGTTTACTCTCTGCCACCTGATCCCAAGTAC
HRV-C_P1 4241 AGTCCTTGGCCTCTAAAGTTATCGCTAGAGGCCTCACTAGTGAGAGCAATGTTTACTCTCTGCCACCTGATCCCAAGTAC
HRV-C_P6 4241 AGTCCTTGGCCTCTAAAGTTATCGCTAGAGGCCTCACTAGTGAGAGCAATGTTTACTCTCTGCCACCTGATCCCAAGTAC

HRV-C_P0 4321 TTTGATGGATACAACCAACAGGAAGTAGTGATCATGGATGATGTTGGCCAAAACCCAGATGGAAAAGATTTGAGTCTATT
HRV-C_P1 4321 TTTGATGGATACAACCAACAGGAAGTAGTGATCATGGATGATGTTGGCCAAAACCCAGATGGAAAAGATTTGAGTCTATT
HRV-C_P6 4321 TTTGATGGATACAACCAACAGGAAGTAGTGATCATGGATGATGTTGGCCAAAACCCAGATGGAAAAGATTTGAGTCTATT

HRV-C_P0 4401 TTGCCAGATGGTGTCAAGCACCGAATTCATAACACCAATGGCCAATCTTGAAGATAAGGGTAGAGCATTTACTAGTGATT
HRV-C_P1 4401 TTGCCAGATGGTGTCAAGCACCGAATTCATAACACCAATGGCCAATCTTGAAGATAAGGGTAGAGCATTTACTAGTGATT
HRV-C_P6 4401 TTGCCAGATGGTGTCAAGCACCGAATTCATAACACCAATGGCCAATCTTGAAGATAAGGGTAGAGCATTTACTAGTGATT

HRV-C_P0 4481 ATGTCATAGCCAGTACAAATATGTCTGTTCTCACACCACCTACTGTCTCATCACCTGAAGCCATAGATAGGAGATTCTTC
HRV-C_P1 4481 ATGTCATAGCCAGTACAAATATGTCTGTTCTCACACCACCTACTGTCTCATCACCTGAAGCCATAGATAGGAGATTCTTC
HRV-C_P6 4481 ATGTCATAGCCAGTACAAATATGTCTGTTCTCACACCACCTACTGTCTCATCACCTGAAGCCATAGATAGGAGATTCTTC

HRV-C_P0 4561 TTGGATTGTGATGTTAAAATCATGCCAGCATATAACACAAATAACCTTCTAAATGTTGCTAAAGCTTTGCTGCCATGCAC
HRV-C_P1 4561 TTGGATTGTGATGTTAAAATCATGCCAGCATATAACACAAATAACCTTCTAAATGTTGCTAAAGCTTTGCTGCCATGCAC
HRV-C_P6 4561 TTGGATTGTGATGTTAAAATCATGCCAGCATATAACACAAATAACCTTCTAAATGTTGCTAAAGCTTTGCTGCCATGCAC

HRV-C_P0 4641 TGATTGCCCAAAACCAGAATTTTATAAACAATGTTGCCCTTTGACATGTGGCAAGGCTTTGATTCTGCAAAACAAAAGGA
HRV-C_P1 4641 TGATTGCCCAAAACCAGAATTTTATAAACAATGTTGCCCTTTGACATGTGGCAAGGCTTTGATTCTGCAAAACAAAAGGA
HRV-C_P6 4641 TGATTGCCCAAAACCAGAATTTTATAAACAATGTTGCCCTTTGACATGTGGCAAGGCTTTGATTCTGCAAAACAAAAGGA

HRV-C_P0 4721 CAAAGGCTAGTTACTCCATCAACACTGTGGTACAACAGCTACGGCAGGAGAAGAAAAACAGGAAATGTGTGGAACACAAT
HRV-C_P1 4721 CAAAGGCTAGTTACTCCATCAACACTGTGGTACAACAGCTACGGCAGGAGAAGAAAAACAGGAAATGTGTGGAACACAAT
HRV-C_P6 4721 CAAAGGCTAGTTACTCCATCAACACTGTGGTACAACAGCTACGGCAGGAGAAGAAAAACAGGAAATGTGTGGAACACAAT
```

```
HRV-C_P0 4801 CTCACTGCCATTTTCCAGGGATTGGGAGATGATTCAACACCAGGATTCATTATAGACCTCCTTAGTGCTTCTAAAGACCC
HRV-C_P1 4801 CTCACTGCCATTTTCCAGGGATTGGGAGATGATTCAACACCAGGATTCATTATAGACCTCCTTAGTGCTTCTAAAGACCC
HRV-C_P6 4801 CTCACTGCCATTTTCCAGGGATTGGGAGATGATTCAACACCAGGATTCATTATAGACCTCCTTAGTGCTTCTAAAGACCC

HRV-C_P0 4881 AAAAGTCATAGAGTTTTGTGAGAAAGAGGGATGGATCAAGCAATCTAGATGCTCAAAGATTGAGCGTGACTTCAACTATG
HRV-C_P1 4881 AAAAGTCATAGAGTTTTGTGAGAAAGAGGGATGGATCAAGCAATCTAGATGCTCAAAGATTGAGCGTGACTTCAACTATG
HRV-C_P6 4881 AAAAGTCATAGAGTTTTGTGAGAAAGAGGGATGGATCAAGCAATCTAGATGCTCAAAGATTGAGCGTGACTTCAACTATG

HRV-C_P0 4961 CACAATACTGCATTAACTGTGTGGGTAGCATCATCTTGATTTTAGGAACTGTTTATGCACTGTATAAGTTGATGTGTATA
HRV-C_P1 4961 CACAATACTGCATTAACTGTGTGGGTAGCATCATCTTGATTTTAGGAACTGTTTATGCACTGTATAAGTTGATGTGTATA
HRV-C_P6 4961 CACAATACTGCATTAACTGTGTGGGTAGCATCATCTTGATTTTAGGAACTGTTTATGCACTGTATAAGTTGATGTGTATA

HRV-C_P0 5041 GCACAAGGCCCCTACACTGGACTACCACAACCAAAAGTAAGAAAACCTGAACTAAGGAAAGCTGTCATTCAAGGTCCAGA
HRV-C_P1 5041 GCACAAGGCCCCTACACTGGACTACCACAACCAAAAGTAAGAAAACCTGAACTAAGGAAAGCTGTCATTCAAGGTCCAGA
HRV-C_P6 5041 GCACAAGGCCCCTACACTGGACTACCACAACCAAAAGTAAGAAAACCTGAACTAAGGAAAGCTGTCATTCAAGGTCCAGA

HRV-C_P0 5121 ACATGAGTTTGGAATGGCATTGATCAAAAGAAACTGCCATATAGCCACAACAGATAGAGGAGACTTTAACCTGCTAGGAG
HRV-C_P1 5121 ACATGAGTTTGGAATGGCATTGATCAAAAGAAACTGCCATATAGCCACAACAGATAGAGGAGACTTTAACCTGCTAGGAG
HRV-C_P6 5121 ACATGAGTTTGGAATGGCATTGATCAAAAGAAACTGCCATATAGCCACAACAGATAGAGGAGACTTTAACCTGCTAGGAG

HRV-C_P0 5201 TGTATGACAACTGTGCAGTGATACCCACACATGCCCAACCAGAGGACACAATTCTTATTGATGGTGTTGAGACAAAAATT
HRV-C_P1 5201 TGTATGACAACTGTGCAGTGATACCCACACATGCCCAACCAGAGGACACAATTCTTATTGATGGTGTTGAGACAAAAATT
HRV-C_P6 5201 TGTATGACAACTGTGCAGTGATACCCACACATGCCCAACCAGAGGACACAATTCTTATTGATGGTGTTGAGACAAAAATT

HRV-C_P0 5281 CTGAAACAGACAATTATCACAGATGAGAACGATGTGGATACAGAGGTGACAATACTGTGGTTAGACCGCAACGAAAAGTT
HRV-C_P1 5281 CTGAAACAGACAATTATCACAGATGAGAACGATGTGGATACAGAGGTGACAATACTGTGGTTAGACCGCAACGAAAAGTT
HRV-C_P6 5281 CTGAAACAGACAATTATCACAGATGAGAACGATGTGGATACAGAGGTGACAATACTGTGGTTAGACCGCAACGAAAAGTT

HRV-C_P0 5361 TAGGGATATAAGAAGGTTCATACCAGATTATCAGAGAGATTGGCAGAATATGCGCCTTGTAACCAATGTGCCCAAATTCC
HRV-C_P1 5361 TAGGGATATAAGAAGGTTCATACCAGATTATCAGAGAGATTGGCAGAATATGCGCCTTGTAACCAATGTGCCCAAATTCC
HRV-C_P6 5361 TAGGGATATAAGAAGGTTCATACCAGATTATCAGAGAGATTGGCAGAATATGCGCCTTGTAACCAATGTGCCCAAATTCC

HRV-C_P0 5441 CCATGCTAGATATAGAGATAGGTGATGTCATACCATATGGAGACATCAATCTGAGTGGAAACCCAACAACCAGACTGCTT
HRV-C_P1 5441 CCATGCTAGATATAGAGATAGGTGATGTCATACCATATGGAGACATCAATCTGAGTGGAAACCCAACAACCAGACTGCTT
HRV-C_P6 5441 CCATGCTAGATATAGAGATAGGTGATGTCATACCATATGGAGACATCAATCTGAGTGGAAACCCAACAACCAGACTGCTT

HRV-C_P0 5521 AAGTATGACTACCCCACGAAACCAGGTCAGTGTGGTGGTGTTATCTTAAATACAGGTAATATAATAGGTATACATGTGGG
HRV-C_P1 5521 AAGTATGACTACCCCACGAAACCAGGTCAGTGTGGTGGTGTTATCTTAAATACAGGTAATATAATAGGTATACATGTGGG
HRV-C_P6 5521 AAGTATGACTACCCCACGAAACCAGGTCAGTGTGGTGGTGTTATCTTAAATACAGGTAATATAATAGGTATACATGTGGG

HRV-C_P0 5601 AGGGAATGGTAGAGTAGGATACTGTGCATCACTAACAAAGAGCTACTTTGCTTCTGCCCAGGGTGAAATAGTCAACAAAT
HRV-C_P1 5601 AGGGAATGGTAGAGTAGGATACTGTGCATCACTAACAAAGAGCTACTTTGCTTCTGCCCAGGGTGAAATAGTCAACAAAT
HRV-C_P6 5601 AGGGAATGGTAGAGTAGGATACTGTGCATCACTAACAAAGAGCTACTTTGCTTCTGCCCAGGGTGAAATAGTCAACAAAT

HRV-C_P0 5681 GCAAAGTACAGGATGTGGGTCTCAAGCCTATCAACACACCAGAACACTCCAAATTACACCCCAGTGTGTTCTATGACATA
HRV-C_P1 5681 GCAAAGTACAGGATGTGGGTCTCAAGCCTATCAACACACCAGAACACTCCAAATTACACCCCAGTGTGTTCTATGACATA
HRV-C_P6 5681 GCAAAGTACAGGATGTGGGTCTCAAGCCTATCAACACACCAGAACACTCCAAATTACACCCCAGTGTGTTCTATGACATA

HRV-C_P0 5761 TTTCCTGGAGAAAAAGAACCGGCTGTACTAACACAGAAAGATCCCAGATTGGAAACTGATTTGAAGACAGCCGTCATGTC
HRV-C_P1 5761 TTTCCTGGAGAAAAAGAACCGGCTGTACTAACACAGAAAGATCCCAGATTGGAAACTGATTTGAAGACAGCCGTCATGTC
HRV-C_P6 5761 TTTCCTGGAGAAAAAGAACCGGCTGTACTAACACAGAAAGATCCCAGATTGGAAACTGATTTGAAGACAGCCGTCATGTC

HRV-C_P0 5841 CAAATACAAAGGGAATGTGAAAATTGAAATGAGTGAGAACATACAGATTGCAGTGGAACATTATTCAGCTCAGCTGTTTA
HRV-C_P1 5841 CAAATACAAAGGGAATGTGAAAATTGAAATGAGTGAGAACATACAGATTGCAGTGGAACATTATTCAGCTCAGCTGTTTA
HRV-C_P6 5841 CAAATACAAAGGGAATGTGAAAATTGAAATGAGTGAGAACATACAGATTGCAGTGGAACATTATTCAGCTCAGCTGTTTA

HRV-C_P0 5921 TGTTAGACATTAACCCAGAACCCATATCTTTGGAACAGGCTGTGTATGGTATGGAAAACCTGGAACCACTCGATCTCACC
HRV-C_P1 5921 TGTTAGACATTAACCCAGAACCCATATCTTTGGAACAGGCTGTGTATGGTATGGAAAACCTGGAACCACTCGATCTCACC
HRV-C_P6 5921 TGTTAGACATTAACCCAGAACCCATATCTTTGGAACAGGCTGTGTATGGTATGGAAAACCTGGAACCACTCGATCTCACC
```

```
HRV-C_P0 6001 ACGAGTGCAGGCTTTCCCTATGTCACCATGGGGGTTAAAAAGAGAGACATTCTGAACAGAAACACAAGGGATGTTACAAA
HRV-C_P1 6001 ACGAGTGCAGGCTTTCCCTATGTCACCATGGGGGTTAAAAAGAGAGACATTCTGAACAGAAACACAAGGGATGTTACAAA
HRV-C_P6 6001 ACGAGTGCAGGCTTTCCCTATGTCACCATGGGGGTTAAAAAGAGAGACATTCTGAACAGAAACACAAGGGATGTTACAAA

HRV-C_P0 6081 ACTTCAACATATGATTGACACCTATGGACTTGATCTACCATATGTGACCTACCTAAAAGATGAGCTTCGGGCTCCTGTTA
HRV-C_P1 6081 ACTTCAACATATGATTGACACCTATGGACTTGATCTACCATATGTGACCTACCTAAAAGATGAGCTTCGGGCTCCTGTTA
HRV-C_P6 6081 ACTTCAACATATGATTGACACCTATGGACTTGATCTACCATATGTGACCTACCTAAAAGATGAGCTTCGGGCTCCTGTTA

HRV-C_P0 6161 AAATAAAAACAGGGAAAACAAGAGCCATTGAAGCAGCTAGCATGAATGACACAGCAAACTTCAGAAGAGTGTTTGGCAAC
HRV-C_P1 6161 AAATAAAAACAGGGAAAACAAGAGCCATTGAAGCAGCTAGCATGAATGACACAGCAAACTTCAGAAGAGTGTTTGGCAAC
HRV-C_P6 6161 AAATAAAAACAGGGAAAACAAGAGCCATTGAAGCAGCTAGCATGAATGACACAGCAAACTTCAGAAGAGTGTTTGGCAAC

HRV-C_P0 6241 TTGTACGCTGGCTTCCATGCAAACCCAGGTATTCTAACTGGGTCAGCTATAGGCTGTGATCCTGACATCTTCTGGTCACA
HRV-C_P1 6241 TTGTACGCTGGCTTCCATGCAAACCCAGGTATTCTAACTGGGTCAGCTATAGGCTGTGATCCTGACATCTTCTGGTCACA
HRV-C_P6 6241 TTGTACGCTGGCTTCCATGCAAACCCAGGTATTCTAACTGGGTCAGCTATAGGCTGTGATCCTGACATCTTCTGGTCACA

HRV-C_P0 6321 ACTATATGCTTCAATGGAAAAACACCTGTTAGTGTTTGATTACACAAATTATGATGGAAGTCTACACCCAGTGTGGTTTC
HRV-C_P1 6321 ACTATATGCTTCAATGGAAAAACACCTGTTAGTGTTTGATTACACAAATTATGATGGAAGTCTACACCCAGTGTGGTTTC
HRV-C_P6 6321 ACTATATGCTTCAATGGAAAAACACCTGTTAGTGTTTGATTACACAAATTATGATGGAAGTCTACACCCAGTGTGGTTTC

HRV-C_P0 6401 AAGCACTTGAACAACTTTTAAACAACTTGGGTTTTCCTGGTGAGTTAGCATATAAACTGTGTAACACAACCCACATATAC
HRV-C_P1 6401 AAGCACTTGAACAACTTTTAAACAACTTGGGTTTTCCTGGTGAGTTAGCATATAAACTGTGTAACACAACCCACATATAC
HRV-C_P6 6401 AAGCACTTGAACAACTTTTAAACAACTTGGGTTTTCCTGGTGAGTTAGCATATAAACTGTGTAACACAACCCACATATAC

HRV-C_P0 6481 AAGGATGAAATATACACAGTGAAGGGAGGAATGCCTTCGGGAATCTCAGGAACCTCCATTTTCAACACAATTATAAACAA
HRV-C_P1 6481 AAGGATGAAATATACACAGTGAAGGGAGGAATGCCTTCGGGAATCTCAGGAACCTCCATTTTCAACACAATTATAAACAA
HRV-C_P6 6481 AAGGATGAAATATACACAGTGAAGGGAGGAATGCCTTCGGGAATCTCAGGAACCTCCATTTTCAACACAATTATAAACAA

HRV-C_P0 6561 CATAATAATTAGGACTTTGGTTTTAGACACATACAAAGGGATTGAATTGGACAAATTAAAAATTGTGGCATATGGTGATG
HRV-C_P1 6561 CATAATAATTAGGACTTTGGTTTTAGACACATACAAAGGGATTGAATTGGACAAATTAAAAATTGTGGCATATGGTGATG
HRV-C_P6 6561 CATAATAATTAGGACTTTGGTTTTAGACACATACAAAGGGATTGAATTGGACAAATTAAAAATTGTGGCATATGGTGATG

HRV-C_P0 6641 ATGTGATAGCTTCCTACCCAGAAGAGCTTGACCCAGCACAGATTGCTGTGTCTGGTCTCAAGTACGGGCTAACAATAACA
HRV-C_P1 6641 ATGTGATAGCTTCCTACCCAGAAGAGCTTGACCCAGCACAGATTGCTGTGTCTGGTCTCAAGTACGGGCTAACAATAACA
HRV-C_P6 6641 ATGTGATAGCTTCCTACCCAGAAGAGCTTGACCCAGCACAGATTGCTGTGTCTGGTCTCAAGTACGGGCTAACAATAACA

HRV-C_P0 6721 CCTGCTGACAAGTCTGACCAGTTTGTGAAAGTCAACTGGGACAATGCCACATTTTTGAAAAGAGGATTCCGTGTGGATGA
HRV-C_P1 6721 CCTGCTGACAAGTCTGACCAGTTTGTGAAAGTCAACTGGGACAATGCCACATTTTTGAAAAGAGGATTCCGTGTGGATGA
HRV-C_P6 6721 CCTGCTGACAAGTCTGACCAGTTTGTGAAAGTCAACTGGGACAATGCCACATTTTTGAAAAGAGGATTCCGTGTGGATGA

HRV-C_P0 6801 GAGATACAGTTTCCTTGTACACCCGGTTTTCCCAGAAAAGGAGATCTTTGAATCGATTAGATGGACCAGAGACCCAAAGA
HRV-C_P1 6801 GAGATACAGTTTCCTTGTACACCCGGTTTTCCCAGAAAAGGAGATCTTTGAATCGATTAGATGGACCAGAGACCCAAAGA
HRV-C_P6 6801 GAGATACAGTTTCCTTGTACACCCGGTTTTCCCAGAAAAGGAGATCTTTGAATCGATTAGATGGACCAGAGACCCAAAGA

HRV-C_P0 6881 ACACTCAAGACCATGTATACTCTTTATGTCTTTTAATGTGGCACAATGGTGAAGAGCCTTACAAGGAGTTCATATCCAAA
HRV-C_P1 6881 ACACTCAAGACCATGTATACTCTTTATGTCTTTTAATGTGGCACAATGGTGAAGAGCCTTACAAGGAGTTCATATCCAAA
HRV-C_P6 6881 ACACTCAAGACCATGTATACTCTTTATGTCTTTTAATGTGGCACAATGGTGAAGAGCCTTACAAGGAGTTCATATCCAAA

HRV-C_P0 6961 ATTAGAAAGACAGAAGTAGGCCGTAGGTTGTATCTTCCACCATACAACCTGCTAGAGCGTACTTGGATTGATAAATTCAT
HRV-C_P1 6961 ATTAGAAAGACAGAAGTAGGCCGTAGGTTGTATCTTCCACCATACAACCTGCTAGAGCGTACTTGGATTGATAAATTCAT
HRV-C_P6 6961 ATTAGAAAGACAGAAGTAGGCCGTAGGTTGTATCTTCCACCATACAACCTGCTAGAGCGTACTTGGATTGATAAATTCAT

HRV-C_P0 7041 CTGAATAGCAATAGTAATGTAGAAATCTACCTGNNNNNNNNNNNNNNNNNNNNNNNNNNNNNNNNNNNNNNNNNNNNNNN
HRV-C_P1 7041 CTGAATAGCAATAGTAATGTAGAAATCTACCTGAGTAGGAGTAGGGTTTTATAAAAAAAAAAAAAAAAAAAAAAAAAAAA
HRV-C_P6 7041 CTGAATAGCAATAGTAATGTAGAAATCTACCTGAGTAGGAGTAGGGTTTTATAAAAAAAAAAAAAAAAAAAAAAAAAAAA

HRV-C_P0 7121 NNNNNNNNNNNNNNN
HRV-C_P1 7121 AAAAAAAAAAAAAAA
HRV-C_P6 7121 AAAAAAAAAAAAAAA
```
