# Supplementary material for: Human respiratory organoids sustained reproducible propagation of human rhinovirus C and elucidation of virus-host interaction
Source: Nat Commun. 2024 Dec 30;15:10772. doi: 10.1038/s41467-024-55076-2 (PMC11686133; doi:10.1038/s41467-024-55076-2)
Supplement: Supplementary file 3 — Reporting summary [file 41467_2024_55076_MOESM3_ESM.pdf]

nature portfolio

Corresponding author(s): Jie ZHOU

Last updated by author(s): Nov 18, 2024

Reporting Summary

Nature Portfolio wishes to improve the reproducibility of the work that we publish. This form provides structure for consistency and transparency in reporting. For further information on Nature Portfolio policies, see our Editorial Policies and the Editorial Policy Checklist.

Statistics

For all statistical analyses, confirm that the following items are present in the figure legend, table legend, main text, or Methods section.

n/a	Confirmed	
☐	☒	The exact sample size (*n*) for each experimental group/condition, given as a discrete number and unit of measurement
☐	☒	A statement on whether measurements were taken from distinct samples or whether the same sample was measured repeatedly
☐	☒	The statistical test(s) used AND whether they are one- or two-sided<br>*Only common tests should be described solely by name; describe more complex techniques in the Methods section.*
☒	☐	A description of all covariates tested
☒	☐	A description of any assumptions or corrections, such as tests of normality and adjustment for multiple comparisons
☒	☐	A full description of the statistical parameters including central tendency (e.g. means) or other basic estimates (e.g. regression coefficient) AND variation (e.g. standard deviation) or associated estimates of uncertainty (e.g. confidence intervals)
☐	☒	For null hypothesis testing, the test statistic (e.g. *F*, *t*, *r*) with confidence intervals, effect sizes, degrees of freedom and *P* value noted<br>*Give P values as exact values whenever suitable.*
☒	☐	For Bayesian analysis, information on the choice of priors and Markov chain Monte Carlo settings
☒	☐	For hierarchical and complex designs, identification of the appropriate level for tests and full reporting of outcomes
☒	☐	Estimates of effect sizes (e.g. Cohen's *d*, Pearson's *r*), indicating how they were calculated

Our web collection on statistics for biologists contains articles on many of the points above.

Software and code

Policy information about availability of computer code

Data collection	No software is used.
Data analysis	GraphPad Prism 9.0 was used for data analysis and plotting the figures.<br>FlowJo software 10.7.1 was used for the analysis of flow cytometry data.<br>MEGA 10.2.3 was used for the phylogenetic tree construction.

For manuscripts utilizing custom algorithms or software that are central to the research but not yet described in published literature, software must be made available to editors and reviewers. We strongly encourage code deposition in a community repository (e.g. GitHub). See the Nature Portfolio guidelines for submitting code & software for further information.

Data

Policy information about availability of data

All manuscripts must include a data availability statement. This statement should provide the following information, where applicable:

- Accession codes, unique identifiers, or web links for publicly available datasets
- A description of any restrictions on data availability
- For clinical datasets or third party data, please ensure that the statement adheres to our policy

The data supporting the findings of this study are available from the corresponding authors upon written request.

Research involving human participants, their data, or biological material

Policy information about studies with human participants or human data. See also policy information about sex, gender (identity/presentation), and sexual orientation and race, ethnicity and racism.

Reporting on sex and gender	Sex- and gender-based analysis was not used in this study.
Reporting on race, ethnicity, or other socially relevant groupings	Race, ethnicity, or other socially relevant groupings were not used in the manuscript.
Population characteristics	HRV-C positive patients who were confirmed by laboratory test. This study only used the patients' swab specimens for virus isolation and analysis. Patients themselves were not involved in any experiments.
Recruitment	The patients were confirmed HRV-C infection in public hospital in Hong Kong.
Ethics oversight	Institutional Review Board of the University of Hong Kong/Hospital Authority Hong Kong West Cluster (UW13-364 and UW21-695)

Note that full information on the approval of the study protocol must also be provided in the manuscript.

Field-specific reporting

Please select the one below that is the best fit for your research. If you are not sure, read the appropriate sections before making your selection.

☒ Life sciences ☐ Behavioural & social sciences ☐ Ecological, evolutionary & environmental sciences

For a reference copy of the document with all sections, see nature.com/documents/nr-reporting-summary-flat.pdf

Life sciences study design

All studies must disclose on these points even when the disclosure is negative.

Sample size	No experiment presented in this study requires caculating sample size .
Data exclusions	No excluded data.
Replication	All infection experiments were repeated at least two independent times. All attempts of replication were successful. Electron Microscopy was performed once, whereas there are at least 5 images for each cell type. The multiplex PCR array was performed one time in triplicate.
Randomization	Not applicable.
Blinding	Blinding is not required.

Reporting for specific materials, systems and methods

We require information from authors about some types of materials, experimental systems and methods used in many studies. Here, indicate whether each material, system or method listed is relevant to your study. If you are not sure if a list item applies to your research, read the appropriate section before selecting a response.

Materials & experimental systems

n/a	Involved in the study	
☐	☒	Antibodies
☒	☐	Eukaryotic cell lines
☒	☐	Palaeontology and archaeology
☒	☐	Animals and other organisms
☒	☐	Clinical data
☒	☐	Dual use research of concern
☒	☐	Plants

Methods

n/a	Involved in the study	
☒	☐	ChIP-seq
☐	☒	Flow cytometry
☒	☐	MRI-based neuroimaging

Antibodies

Antibodies used

Antibody	Supplier	Catalog number	Clone name	Lot number
Rhinovirus VP3 Antibody	Invitrogen	MA5-18249	G47A	YD3803932

Anti-FLJ23834 antibody	Abcam	ab121337	polyclonal	1016309-2
Anti-CDHR3 antibody	Sigma	HPA011218	polyclonal	000006575
Anti-β-Tubulin IV antibody	Sigma	T7941	ONS.1A6	088M4793
Anti-beta IV Tubulin antibody	Abcam	ab179504	EPR16776	GR252919-6
Mouse IgG	Abcam	ab91353	B11/6	GR3327311-3
Rabbit IgG	Abcam	ab172730	EPR25A	GR3284310-8

Validation

The application of commercial antibodies for IF staining has been validated by the providers.

Plants

Seed stocks

No seed was used in this study.

Novel plant genotypes

No plants were used in this study.

Authentication

No plants were used in this study.

Flow Cytometry

Plots

Confirm that:

☒ The axis labels state the marker and fluorochrome used (e.g. CD4-FITC).

☒ The axis scales are clearly visible. Include numbers along axes only for bottom left plot of group (a 'group' is an analysis of identical markers).

☒ All plots are contour plots with outliers or pseudocolor plots.

☒ A numerical value for number of cells or percentage (with statistics) is provided.

Methodology

Sample preparation

Organoids were dissociated into single cells with 10mM EDTA at 37°C for 30-60 minutes and fixed with 4% PFA for 30 minutes at room temperature. Cells were permeabilized with 0.1% Triton X-100 for 5 minutes at 4°C, and stained with primary and secondary antibodies.

Instrument

Agilent NovoCyte Quanteon analyzer

Software

FlowJo software Version: 10.7.1 is used for the analysis of flow cytometry data.

Cell population abundance

The cells were not sorted in our flow cytometry analysis.

Gating strategy

1) FSC-A and SSC-A were used to gate for the bulk population of cells, 2) FSC-H and FSC-W or SSC-H and SSC-W were used to gate for the single cells, 3) CDHR3 positive populations were determined and gated compared to Isotype IgG controls. 4) VP3 positive populations were gated compared to Mock-infected cells.

☒ Tick this box to confirm that a figure exemplifying the gating strategy is provided in the Supplementary Information.
